# Book Reviews

**Published:** 1803-03-01

**Authors:** 


### CRITICAL ANALYSIS

OF THE

### RECENT PUBLICATIONS

ON THE

### DIFFERENT BRANCHES OF PHYSIC, SURGERY, AND MEDICAL PHILOSOPHY.

---

### *Mr. John Bell's Principles of Surgery.*

[ Concluded from p. 182 of our last. ]

"Ask a young man who has studied his profession faithfully," says our author, "what he would do with a fractured limb? he cannot tell. Ask the same question of one who has practised his profession long, who has moreover practised it well and sensibly. He can hardly tell how he himself is accustomed to manage a fractured limb; he has no rule nor settled method. . . . . . Ask the man of book and study, what have been the doctrines of the old, or the actual improvements of the modern surgeons—he also is at a loss." . . . &c. &c. Can the author really mean to assert that we have no rules nor regular method of practice in the treatment of fractured limbs, that we are unacquainted with the nature of callus, with the gradual process of ossification by which nature procures the reunion of a broken bone? and do we still suppose, with the older surgeons, that callus is "an inorganic concrete poured out from the extremities of the ruptured vessels, which sets or or hardens like stucco and Paris plaster into the consistence of bone?" This is not the only instance that we have met with in the volume before us of undervaluing or neglecting to relate the actual practice of modern surgery; the author is abundantly liberal in recording the errors of antient practitioners, (and it is but justice to add, his strictures on their operations and opinions abound with strong sense and acute reasoning) but Mr. Bell must know that the respectable names of Sharpe, Gooch, or Aitken, no longer carry with them undisputed authority; and the juster pathology and improved practice which in many instances have been adopted subsequent to these ornaments of their profession, are not now to be announced to us as discoveries or untried improvements.

The author considers the important subject of fracture under the following heads:

1. The opinions, prejudices, operations, and daring practices of the older surgeons. These we shall not here enumerate. They consist of theories concerning the nature of callus, and plans of treatment during its formation, abounding with false notions of the pathology of broken bone, and dangerous errors in practice. This was the age for a multiplicity of inventions, many of them (mechanically considered) highly ingenious, for the regulation of the growth of callus, for confining it with tight bandages and straps, buckling it into shape, moulding it by compresses, and binding it over to good behaviour. The author here explains the more accurate idea of the ossifying process and growth of callus which modern physiology has introduced, and we may add, what several of our readers may not be aware of, that he shews very clearly that the accident of breaking the bone a second time during the union of the first fracture, is sooner repaired, and the broken ends reunite in a shorter period than at first.

2. The second head contains the definitions of the various species of fracture, which is clear and sufficiently comprehensive for practical purposes.

3. The third head (which forms a distinct chapter) comprehends a subject of great importance, namely the various accidents of the hip joint, its luxations, fractures, caries, and all the formidable consequences of these complicated injuries; formidable on every account, both from the intricacy of the parts, the resistance which their natural conformation so often opposes to the curative process, and the difficulties which press upon the surgeon in every shape in these unfortunate accidents.

The author begins this chapter with an excellent sketch of the general anatomy of the hip joint; of the parts of which it is composed, their mutual connexions and tendencies to injury; and this is a species of descriptive *topography*, in which we think him at all times peculiarly happy. It is here, as in other parts of the work, illustrated with perspicuous drawings. This introduces the subject of luxation of the femur, a subject which will be read with interest. From luxation he proceeds to fracture, and treats very fully on the calamitous accident of fracture of the neck of the femur.

Of this accident he lays down two distinct varieties, which are followed by very different consequences. In the one, the injury done to the part is no more than the simple fracture of the neck of the bone; *the capsule* especially *remains entire*, and the limb is but little shortened. In the latter case, owing to the originally superior violence of the injury, or the frantic efforts of the patient after the accident, the broken end of the bone is driven through the lacerated capsule, the muscles are displaced from their beds upon the back of the haunch bone, and the limb is shortened full four inches. The consequences of each injury differ very materially, though in both they are equally calamitous. In the former, the whole articulating process of the bone lies within the capsule, and the broken ends often unite indeed, but only by a loose, and, as Dessault observes, by a kind of *fibrous production;* and in no instance is the adhesion sufficiently firm to allow of the future use of the limb. In the second case, that is, where the broken end of the bone is driven with violence through the capsule, high inflammation takes place, an abundance of callus will be produced, but without that nice adaptation which obtains in fractures of the lower limbs, and the fractured bones will unite in a clumsy bulky mass of callus, which generally renders the limb burdensome and almost as useless as in the former instance. The diagnosis of luxation concludes this chapter, and it is illustrated by drawings of the luxated bones *in situ*.

Fracture of the thigh bone occupies the next chapter; and in this accident it is well known, that the contraction of the muscles and shortening of the limb, oppose the most formidable obstacles to a perfect cure. To counteract the muscular contraction, a profusion of machines have been contrived from the earliest times, many of which would not disgrace the invention of an inquisitor, and the author presents us with drawings and descriptions of a great number of them. The ill success of these complicated machines has obliged the contrivers successively to throw them aside, and to resort to a simpler and gentler practice.

The concluding chapter of the subject and the volume, is one that will be perused with much interest, as it contains a variety of important directions and rules for the management of simple, compound, and gun-shot fractures, deduced from the practice of the best surgeons, and from the doctrines explained in the preceding part of the work. It is more practical than the former part, and comprehends many excellent observations. The directions which the author gives, are full, precise, and yet simple, so that the reader cannot but understand both the purport of the rules which are laid down and the reason for their adoption. We shall not enter into all the particulars of this comprehensive chapter, but shall only observe, that after treating of all the varieties of simple fracture, he proceeds to the compound, to the dreadful laceration caused caused by gun shot, and concludes with the question of the propriety of amputation; a question which can never be determined by general rules, but must be considered with all its relations to general state of health, to climate, period of life, and especially to the situation of the patient and the degree of attention and medical care which he may be able to command.

We shall just add, that the author, with very laudable attention to some of the most important stations in which surgical skill is required, takes frequent oppportunities of introducing practical directions for the information of the student who devotes his time to the army or navy service; a service which possesses every claim to the collective contributions from the skill and experience of the whole profession, and a school which has sent forth many of the brightest ornaments of the art of surgery.

---

*Observations on the Constitution of Women, and on some of the Diseases to which they are more especially liable. By* Sayer Walker, M. D. *of the Royal College of Physicians, London, Physician to the London Lying-in Hospital.* London, 1803. pp. 208. 8vo.

The peculiarities of the female constitution, and the treatment of diseases to which the sex are more immediately liable, are subjects which have been so often and so well written on, that we can hardly expect to find any new observations in a work like the present. Nevertheless, as several points of practice, and those of no small importance, are still under discussion with the medical public, we are glad to receive the results of the observation of so experienced a practitioner as the author of the Treatise before us. The public opportunities for enquiry which he has enjoyed are very extensive (as he observes in the preface) having been in the habit of regular attendance on all the duties of the medical department, in the City of London Lying-in Hospital, for nearly nine years, during which period several thousand women have been admitted, the care of whom and of their children, in every disease under which they have laboured, has devolved upon him. The more immediate object of the present publication appears to be that of correcting some popular and vulgar erroneous opinions which are prevalent among the sex and their female advisers. The most prominent of these is the use of the *heating regimen* during the puerperal state; of cordials and volatiles, with a view to support and strengthen the patient under hæmorrhages that occur at different periods. Every practitioner that has attended to the management of females, in every rank of life, and more especially among the lower class in this metropolis, must be convinced of the extensive prevalence of this regimen, and of the mischief which it often produces. Errors of this kind, supported by the authority of the physician, and fostered by natural inclination, take a deep root, and spread widely and rapidly. To oppose them by the voice of cool and prudent advice, is an arduous but honourable contest; and it is probably on this account that the author has compressed his observations into a small compass, and rendered the language, and method of treating the subject, in some degree popular, that sensible and well-informed females may themselves contribute to an object in which they are so much interested.

The arrangement followed in the Treatise before us is simple and perspicuous. The subject is introduced with a sketch of the peculiarities of the female constitution, compared with that of the stronger sex, producing the tendency to disorders of increased irritability, and a habit of body and mind susceptible to every impression. The important period of menstruation next engages the author's attention; and, connected with it, the diseases of chlorosis, and suppression of the menses. In describing the latter, the following important remark is introduced; "A very common practice with patients labouring under this complaint, is to fly immediately to the use of the most forcing medicines (as they term them) that may be presented to their notice, in a public advertisement, as specific remedies for the particular disease under which they labour." He adds, "Too great a solicitude on the part of the parents or friends, to procure the appearance of the menses at a certain time of life, has led to the premature use of those very remedies which, under different circumstances, might have been useful."

The danger which the author apprehends (often we fear with too much reason) is an inflammatory state of the body, produced by the injudicious use of such remedies, and hæmorrhages, not the spontaneous and salutary efforts of Nature, but the consequences of the artificially increased activity of circulation.

The general appearance of a chlorotic patient seems to indicate that the constitution is not yet prepared to produce the menstrual discharge; and therefore, he argues, much mischief may be done by rashly attempting to anticipate a process of Nature, which can be only salutary when it is spontaneous. To prepare the constitution for this process, by improving the general health and activity of body, is very different from exciting it by violent medicines, which bring on a comparative precocity of the uterine system, often at the expence of the rest of the constitution. The particular danger which the author views in such a practice is that of consumption, the diagnosis of which, when combined with chlorosis, every practitioner knows to be attended with peculiar difficulty.

The subjects of menorrhagia and fluor albus, compleat the sketch of female diseases in the earlier part of life. In fluor albus, he notices its very early appearance in some children, long before the age of puberty, which often is so considerable as to require some attention.

The second section, contains the history of the diseases of advanced life, that is to say, the various inconveniences, and sometimes serious diseases to which women are liable during the period of the cessation of the menses. Of these, the most important are uterine uterine hæmorrhage, and schirrus or cancer. The succinct manner in which the author treats of these disorders, precludes any very interesting description or important pathological investigation.

The diseases that occur during pregnancy are next considered, in the same concise but comprehensive manner; they are, nausea, heart-burn, costiveness, derangement of the urinary functions; œdematous enlargement of the lower extremities, cramp, spasm of the stomach and bowels; convulsions, hæmorrhage, and premature labour. Some confusion is occasioned by this extreme brevity, particularly on the subject of parturient convulsions and epileptic paroxysm. It does not appear whether the author considers them as separate affections, to be treated equally by blood-letting, and the antiphlogistic plan. More attention is very properly bestowed on the important subject of uterine hæmorrhage, as being by far the most dangerous accident to which parturient women are liable.

The diseases which occur after parturition naturally follow, and occupy the remainder of the volume. The inflammation of the breast and the milk fever, require the peculiar attention of the practitioner. The local symptoms are thus described. "The patient first complains of a sense of fulness and uneasiness in her breast, attended with a weight and pressure, which naturally leads her to place her hand underneath, with a view to support it. In some cases this is succeeded by uneasiness in the axilla, or by a hard lump in some part of the breast, which is particularly susceptible of pain upon pressure. If the inflammation continue, it will assume a florid colour, which is gradually diffused over the whole of the organ. In a little time, if the disease be not arrested in its progress, some part will appear more prominent than the rest, and a throbbing pain will be felt; in this way it will go on till suppuration takes place, when, as the matter contained increases and the skin becomes thinner, it will be felt by the finger, and in a little time will be discharged from the most dependent part of the tumour." The usual uncertainty with regard to topical applications here appears; in the incipient stage, the author observes, the use of some cooling lotion may be very proper: emollient fomentations and cataplasms may also be applied with advantage; but no precepts for the regulation of these very opposite applications are attempted. Where the child does not suck, he recommends as a means of checking the flow of milk, to place in the axilla a piece of cotton dipped in an embrocation, consisting of aqua ammonia, one part, with sp. vin. camph. three parts.

It might be expected that the subject of puerperal fever should engage the attention of a practitioner, whose opportunities for observing this disease have been considerable; and accordingly, we have a fuller history of it than of most of the other female maladies. The description is clear and accurate, and the appearances on dissection are given in this case, in a few sentences. The mode of practice recommended is the antiphlogistic, to as great an extent as can be pursued with safety.

Puerperal mania follows, and the author is disposed to make a distinction between simple alienation of mind in the puerperal state and active phrenitis. The cause of the latter dreadful disorder is involved in obscurity, and the best method of treatment scarcely indicated. The volume concludes with some remarks on the phlegmatia dolens; a disease which has of late attracted some attention.

The reader who expects to receive in this little volume, from the physician to a large and excellently conducted charity, any new observations or interesting discussion, concerning some of the more important or less common of the female diseases, will find himself entirely disappointed; but as a neat, brief sketch of a very extensive subject, and a summary view of the actual practice of the present day, cautiously, and we might almost say, *anxiously* divested of theoretical discussion on controverted points, it will be well received by those who want leisure or ability to pursue the subject more at large, and for any purpose of extensive utility.

---

*Extract from an Account of Cases of Typhus Fever, in which the Affusion of Cold Water has been applied in the London House of Recovery. By* W. P. Dimsdale, *Physician to the Institution.* London, printed for the Society for bettering the Condition of the Poor.

The valuable practice of cold affusion, lately revived by Dr. Currie, and considered in so truly a philosophical manner in his excellent work on Fever, must often have come under the notice of all our readers; but its progress is still slow in this metropolis, and in many other parts of the kingdom. It is therefore, with great satisfaction that we observe the experiment (if indeed it can still be called *experiment)* pursued in the London House of Recovery, with so much vigour and such perfect success. Dr. Dimsdale, in these few pages, clearly and briefly relates twelve cases of perfect typhus fever cured by this simple remedy; and the perspicuous simplicity of the narration gives the strongest internal evidence of its fidelity. *(See the work itself, p.* 206 *of this Number.)*

---

Dr. Trotter's Medicina Nautica,

[*Continued from p.* 185—187 *of our last Number.*]

Dr. T. presents us with a Sketch of the contents of this third volume in his Introduction. "The present volume," says he, "I trust, will be found to be not inferior to either of the former. The dismissal of the hospital ship cut off much of my communication with the fleet; and in some parts I appear rather the historian of the afflictions of the sick, than their Physician. Lord Bridport ordered all the stores of that ship, which had been the pride of our service to complete, to be landed, without ever consulting me, whether any thing ought to be reserved, lest the fleet came to action, or for other eventual malady. The consequence has has been, that the number of deaths at sea has been very great beyond the preceding seasons, and the sick deprived of all the comforts which the Admiralty, under Earl Spencer, had so bountifully granted. Fresh meat and vegetables, it is true, were liberally supplied in 1801, off Ushant; but these are only a few out of many delicacies with which our hospital was stowed. My sentiments on these subjects are not fashioned to the opinions of any set of men; but, what I conceive to be better, they are the language of British benevolence, and the practice of a physician who, amidst all his foibles, has not been known to discover the spirit of temporizing. Studies similar to these procured me the appointment which I have the honour to hold; they have supported me through much bodily fatigue and mental exertion; and they shall attend me through this last of my labours, in the naval service of my country. Many improvements of acknowledged utility have originated with me, and many have been suggested that others will have to accomplish. In this, as in my former volumes, many official representations are introduced for the information of our successors. In the event of a long peace, they might otherwise be lost to the public. The navy at this moment abounds with young men of the medical profession, who are earnestly attached to its studies, and ardent after improvement. These gentlemen, at some future day, will occupy the posts of honour in the department; but the same prejudices that have operated against me as a beginner of correction, are not likely to be opposed to them in the same degree. And in this manner will be attained, in fifty years, that perfection of medical duty and arrangements, which, I think, might be brought about in as many days. Had I possessed the power, as I do the inclination, this task should not have been left to others.

"Medical readers will be astonished to see, at this period of a triumphant navy, so many abuses left to prey upon health, and that so little desire after improvement should be discovered. But it is with the health of the public as it is with the individual, the value of it is not thought of till it is lost. I have therefore inserted the copy of a *Letter* to the present First Lord of the Admiralty, "*on meliorating the encouragement to medical officers.* It exhibits in a concise view a radical method of reform; and, I hope, will be found equally consistent with the benefit of service and that of the surgeon. It is at least proper that long experience on this subject, as well as others, should be recorded, till a disposition shall be manifested to reduce it to practice.

"Such *occurrences* relating to health as are any how interesting, are detailed, as in the former volumes. Some things, it may be remarked, might have been better omitted: '*but my pen guides me, I guide not it.*'

"The subjects of *Contagion and Typhus* are comprized under one article; which enables me to offer frequent practical remarks, as suggested by matter communicated by the surgeons. These will be

 found found rather to enliven the narrative than to interrupt it. This head affords many shocking examples of the horrors of the impress service; would that I possessed eloquence sufficient to convince statesmen of its iniquity; for the facts are palpable! If it is wanted to subdue effectually that spirit of insubordination and revolt, which has so repeatedly appeared this war, it must be by finding *another method* for manning the navy. Make the service of your seamen voluntary, and tumult will be at an end.

"Some *Thoughts on preventing the Plague* follow next. They were written three years ago, when that disease was much the subject of conversation in this country.

"A distinct article is now allotted to the *ventilation of ships.* This, I hope, will be useful to officers; for it is of the first importance in preserving the health of a ship's company. It might have been extended to a much greater length, in explaining theory; but for practical utility it will be deemed sufficient, as it fully unfolds the causes that generate foul airs.

"The *Small Pox* continues to excite our vigilance; and the *Cow Pox*, that first blessing from the hands of medicine, is now received among sea diseases, on a prophylactic plan. I have only to regret that I could not accomplish my purpose on this business.—*Catarrh*, *Pneumonia*, and *Ophthalmia*, are connected in one article.

"The subject of *Phthisis* is a new discussion in *Medicina Nautica.* If the treatment of the disease receive no advantages from our animadversions, the history of it is enlarged. The causes which have rendered it frequent in the Channel, especially in 1800, are not calculated to raise pleasurable feelings. But what human being can brave variety of affliction equal to a British seaman!

"Under the term of *Spasmodic Affections*, I have introduced various complaints of the dyspeptic, hypochondriacal, and nervous kind. These diseases are by no means uncommon in a man of war at all times, but they have been very frequent of late. It is the more necessary to guard the inexperienced surgeon, as they are often mistaken for complaints that require a different mode of treatment.

"We continue to accumulate such facts on the history of *Scurvy*, as may still improve the method of prevention and cure. In this, as in some other subjects, we have looked beyond the mere means of cure; our views are directed to preserve the strength and vigour of muscular action, for the purpose of enterprize.

"Some valuable selections are made from the communications of our numerous correspondents, as incidental to our plan. Parts of these are from the Mediterranean, which station has been the scene of considerable activity during the operations in Egypt and Malta.

"For the first time, we offer the plan of a *Sick Berth*, which is to be considered as the hospital of a ship of the line. I have also given a method of providing a *Diet for the Sick*, that is much superior superior to any thing of the kind that has ever appeared in the navy. A mixture of regret accompanies this part of my work, that I have not been able to make this improvement general throughout the fleet and service at large. But it is necessary to save it from the wrecks of time.—*Sea Sickness* has also, for the first time, met with our notice.

" The history of the *Malignant Ulcer*, by the additions of the present Volume, is rendered very full. I wish I could say that they have pointed out any successful method of cure.

" The contents of this Volume, in different parts, are so much a sequel to what I have written before, that it is necessary to remind the readers of it, lest they should form opinions partially. For instance, in the article Contagion, where I have exposed the bad effects resulting from the impress service, it will be seen in the first Volume, that I had suggested means of guarding against all this calamity as a source of disease. In every disquisition it will be easily perceived, that the author has had one object in view, to which all others have been secondary.

" From the Interest which I have taken in the cause of medical officers from my first becoming an author, the subject naturally recurs to me, in bringing this last of my labours to a conclusion. I must therefore invoke the different tribunals of science and literature, as they may honour this Volume with a perusal, that they would well consider the arguments which I have produced in favour of increased encouragement to these gentlemen. Physicians to fleets are equally subject to a narrow establishment with surgeons; and one of the most painful consequences of a small income, must be the inability to keep pace with the progress of medical science in the purchase of new books. With this last wish in behalf of a body of men to whom the public owes much, I now conclude; and thus, my beloved Navy! farewell!"

---

*A New Medical Dictionary containing a concise Explanation of all the Terms used in Medicine, Surgery, Pharmacy, Botany, Natural History, and Chemistry. Compiled by* JOSEPH FOX, M. D. *late Physician to the London Hospital. Revised and augmented by* THOMAS BRADLEY, M. D. *Physician to the Westminster Hospital, &c.* 12mo. London, 1803.

THE peculiar merit of this very small and portable dictionary, consists in the enumeration of *all the terms* employed in the several sciences connected with Medicine and Surgery. The explanations are given in a concise manner, under each term, without reference to distant parts of the book, and the most useful species and varieties are repeated under each genus, by which we think the memory will be much assisted and relieved. The Editors appear to consider this dictionary also, as a very useful means of furnishing students and medical readers with a valuable index to their common place book, or a means of reference to any author who has suggested useful hints or improvements on medical subjects.

*Observations on Dr.* PEARSON'S *Examination of the Report of the Vaccine Pock Committee, of the House of Commons, concerning Dr.* JENNER'S *claim for Remuneration. By* THOMAS CREASER, *Member of the Royal College of Surgeons, London.* 8vo. pp. 90. Bath, 1803.

THESE observations appear to us to be candid, fair, and well becoming the great respectability of the author. But as we have already given our opinion on this subject at length; and as we believe the mind of every medical practitioner is made up respecting it, we shall not enter into a particular analysis of the pamphlet at present.

---

*An Essay on the Medical Application of Electricity. By* JOHN BIRCH, *Esq. &c.* 8vo. pp. 57. London, 1803.

WE think the various applications of Galvanism to medical purposes, which have been made during the last months, require that we should revive and support an attention to Electricity in *general*, as a powerful mean of preserving and restoring health. Mr. B. explains his motives for publishing this pamphlet in the following terms: "That those opinions on the medical application of Electricity, illustrated by cases which were published some years since by the late ingenious Mr. George Adams, have been considered as useful, must appear not only from the demand which has been made in England for the book, but also from its having been translated into the German and Italian languages. I am now solicited to revise and reprint that little work by itself, which I most willingly undertake, in order to promote the medical application of electricity.

"Experience is the test by which we can judge of a proposition; and repeated facts, well authenticated, carry conviction with them. It is now upwards of twenty years that I have unremittingly pursued this point of study, with the assent and approbation of some of the ablest practitioners. The late Doctors Heberden and Warren, Mr. Else, Mr. J. Hunter, and Mr. Gunning, were witnesses to several of the cases here mentioned: to these cases I could make large additions, if I thought it necessary; but additions are needless, as it must be evident, since the electric power has efficacy sufficient to cure diseases of such consequence, it will not fail in those of inferior degrees."

---

*An Account of the Discovery and Operation of a New Medicine for Gout;* 8vo. pp. 194. London. 1803.

IN this age of parsimony, respecting medical remuneration, we cannot be surprised to see a gentleman, who believes he has discovered a remedy for one of the greatest affiictions of advanced age and affluence, come forward with caution, and endeavour to secure cure some adequate reward for his labours and anxiety. Mr. W. assures us that he will *publish* his medicine as soon as its efficacy is established, under the inspection of medical practitioners who may be thought competent to decide on its safety and merits.

